# The Past, Present, and Future Distribution of *Sargentodoxa*: Perspectives From Fossil Record and Species Distribution Models

**DOI:** 10.1002/ece3.71831

**Published:** 2025-07-20

**Authors:** Xuanqi Liu, Huasheng Huang, Xia Meng, Minqiao Li, Zeyu Qin

**Affiliations:** ^1^ School of Geography and Planning Sun Yat‐sen University Guangzhou China; ^2^ Carbon‐Water Observation and Research Station in Karst Regions of Northern Guangdong, School of Geography and Planning Sun Yat‐sen University Guangzhou China

**Keywords:** biogeography, climate change, glacial refugia, habitat suitability, Lardizabalaceae, machine learning, random forest model

## Abstract

Global climate change is a critical factor influencing biodiversity and ecosystem stability by altering the suitable habitats of many species. *Sargentodoxa cuneata* is an endemic and relict plant species in China. Identifying its suitable habitats across different periods and glacial refugia helps explain how *S. cuneata* survived Quaternary climate fluctuations, which is crucial for informing its future conservation. However, long‐term tracking of its distribution and systematic description of biogeographical evolution remain scarce. Here, we compare 10 species distribution models to assess their predictive performance. Ultimately, we apply a random forest model to simulate the suitable habitats of *S. cuneata* under past, present, and future climate scenarios and integrate fossil records to analyze its biogeographical history. We find that *S. cuneata* once had a much broader distribution, likely originating in North America, with subsequent migration to Europe and Asia, and its range has gradually contracted, now primarily persisting in East Asia. It is currently distributed mainly south of the Qinling‐Huaihe Line in China, particularly in mid‐ and low‐altitude mountainous regions with abundant precipitation and moderate temperatures. During the Last Glacial Maximum (LGM, ~22,000 years ago) and Mid‐Holocene (MH, ~6000 years ago), its suitable habitat contracted significantly, with extremely suitable areas nearly disappearing due to colder climate. Glacial refugia are identified in three mountain ranges within Central and South China. Model simulations under two different climate scenarios suggest that while the total suitable habitat of *S. cuneata* may expand, extremely suitable areas could decline, with a northward expansion and southern contraction. This study will provide insights into the long‐term impact of climate change on relict plant species and contribute to a better understanding of the evolutionary history of East Asian flora.

## Graphical abstract

We traced the biogeographical history of *Sargentodoxa cuneata*. Three mountain ranges are likely to have served as glacial refugia for *S. cuneata* in the Last Glacial Maximum. Future distribution of *S. cuneata* is expected to expand northward and contract southward.

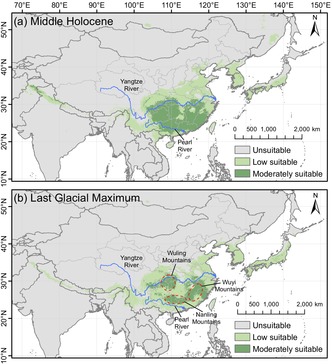

## 1 Introduction

Over the past few decades, the global average temperature has steadily risen, accompanied by an increasing frequency of extreme climatic events. This has significantly impacted ecosystem stability and species adaptive capacities. Since the Quaternary period, glacial‐interglacial cycles have driven habitat shifts in many extant species across the Northern Hemisphere (Fu and Wen 2023) and have also led to genomic divergence (Guo et al. 2023; Yin et al. 2021). Climate change not only reshapes habitat suitability but also contributes to habitat loss, population declines, and even extinction (Wiens and Zelinka 2024). In the face of escalating climate change, species geographical distribution patterns are undergoing profound transformations. Accurately predicting these distributional shifts and glacial refugia is essential for biodiversity conservation, biological resource management, and sustainable development (Wang et al. 2023).

The species distribution models (SDMs), also known as ecological (or environmental) niche models (ENMs), have become a fundamental tool for assessing the influence of environmental factors on species distributions. The SDMs have been widely applied in tracing ecological indication and biogeographical history (e.g., Kang et al. 2025; Qi et al. 2012; Tang et al. 2017; Zhao et al. 2021). They estimate potential suitable habitats for species across different temporal scales by modeling the relationship between known species occurrences and environmental variables (Elith and Leathwick 2009). The SDM algorithms that have been commonly used include maximum entropy model (MaxEnt) (Phillips et al. 2006), random forest (RF) (Breiman 2001), generalized linear models (GLM) (Nelder and Wedderburn 1972), and generalized additive models (GAM) (Hastie and Tibshirani 1987), among others. The selection of the most appropriate modeling approach depends on the characteristics and objectives of the study (Hao et al. 2020; Li and Wang 2013).

Glacial refugia refer to regions that provided climatically suitable conditions for species persistence during global glacial periods, such as the Last Glacial Maximum (LGM, ~22,000 years ago) (Médail and Diadema 2009). These areas are sometimes also referred to as climate refugia (Hampe et al. 2013). Plants, highly sensitive to climatic fluctuations, rely on refugia not only for survival but also as centers of genetic differentiation and adaptive evolution. Consequently, glacial refugia play a critical role in shaping evolutionary processes, maintaining biodiversity, and influencing present‐day species distribution patterns (Tang et al. 2018). During glacial periods, many species experienced severe range contractions or even local extinctions due to temperature declines and ice sheet expansion. However, certain regions with complex topography and diverse microclimates, such as Southwest China (Tang et al. 2018), the Mediterranean Basin (Petit et al. 2003), and the Himalayas (Singh et al. 2020), served as crucial refugia for subtropical and temperate plants. These areas not only facilitated species survival during glacial episodes but also acted as postglacial centers of recolonization, shaping contemporary distribution patterns. By using the SDMs, we can delineate the current suitable habitat range of a species, reconstruct its historical distribution patterns under past climatic conditions, and thus identify its glacial refugia. Additionally, the SDMs can be used to predict potential habitat shifts under future climate change scenarios, and this provides evidence for the development of effective conservation strategies.

**FIGURE 1:**
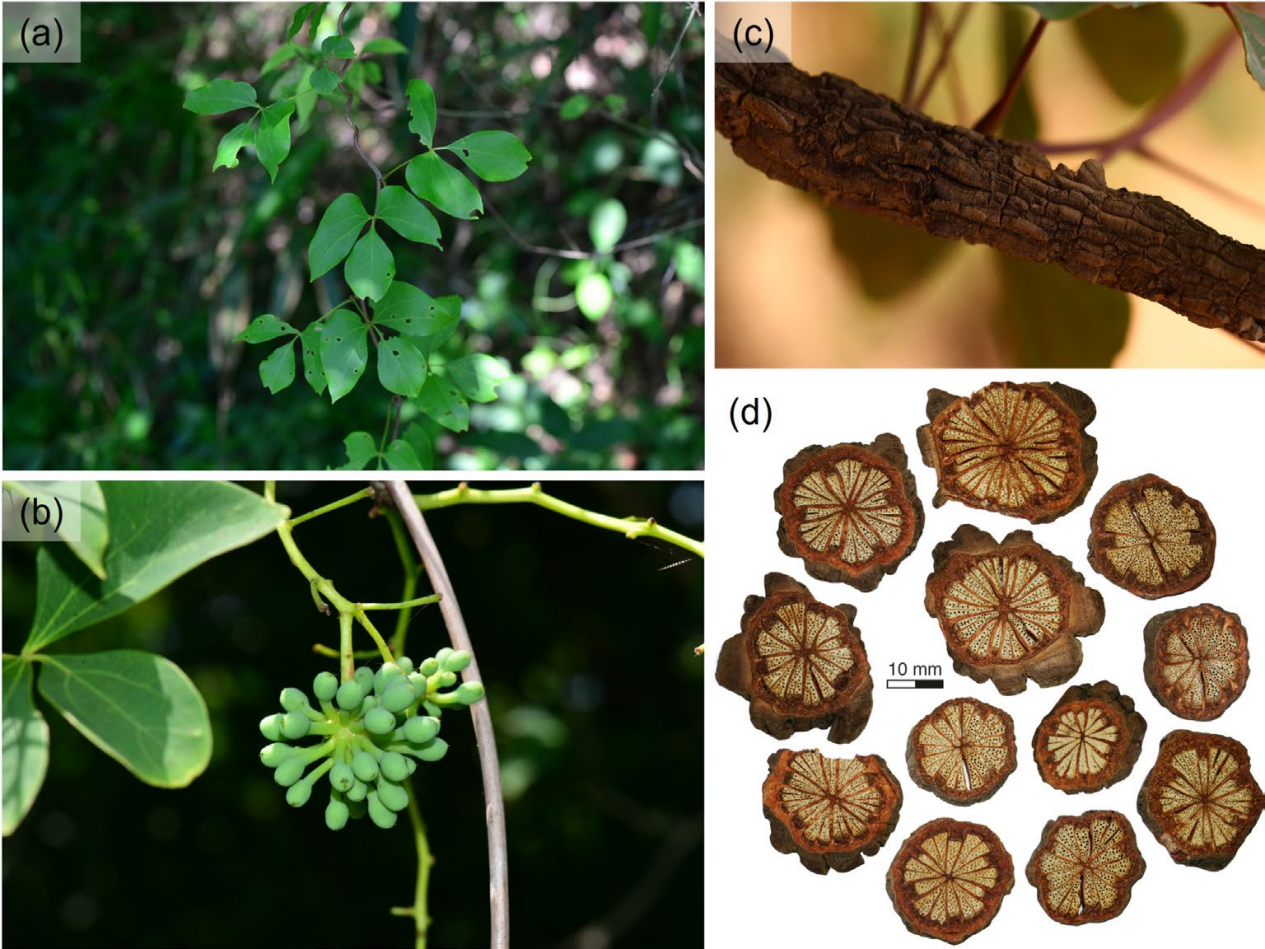
Leaves (a), fruits (b), rattan (c), and medicinal slices (d) of *Sargentodoxa cuneata*. The medicinal slices are made from the cleaned and dried stems. Photos (a–c) courtesy of Dr. Renbin Zhu, (d) taken by Dr. Huasheng Huang.

The genus *Sargentodoxa* Rehder & E.H.Wilson belongs to the family Lardizabalaceae and is a monotypic genus containing only *Sargentodoxa cuneata* (Oliv.) Rehd. et Wils (Figure 1). *S. cuneata* is a relict species endemic to China and is primarily distributed in subtropical regions, with occasional occurrences in northern Laos and Vietnam. It commonly grows in well‐lit open forests, forest edges, and shrublands on mountain slopes at elevations of 100–1000 m (Chen and Tatemi 2001). *S. cuneata* holds significant medicinal value (Zhao et al. 2014). Its bioactive compounds exhibit anti‐inflammatory, antibacterial, and antitumor properties, which are helpful for treating various diseases, particularly arthritis and sepsis (Zhang et al. 2021). Consequently, the sustainable utilization and conservation of this species have attracted considerable attention. Currently, research on the medicinal properties of *S. cuneata* is relatively abundant (Wang, Zhang, et al. 2024; Xu et al. 2024), while studies on its spatiotemporal distribution remain limited (Tian et al. 2015).

Climate change is expected to have profound impacts on the spatiotemporal distribution of *S. cuneata* . Fossil evidence indicates that *S. cuneata* once had a broader distribution (across the globe) over the past several million years than at present. However, following large‐scale extinctions in the Americas and Europe, its habitat is now restricted to certain regions of East Asia (Manchester et al. 2009; Zhou and Arata 2005). Analyzing long‐term biogeographical dynamics using fossil records can enhance the accuracy and interpretation of short‐term distribution predictions in the context of ongoing climate change. The identification of historical glacial refugia for *S. cuneata* can shed light on why its current distribution is limited to China and offer insights into its potential adaptability to different climatic conditions. Furthermore, these refugial regions may still serve as contemporary genetic diversity hotspots for *S. cuneata* . Recognizing and protecting these areas is crucial for preserving genetic resources and enhancing the species resilience to future climate change.

Although *S. cuneata* is a relict species of significant ecological and medicinal value, studies on its suitable habitat distribution remain scarce. Therefore, this study aims to address the following key questions: How does the suitable habitat of *S. cuneata* change from the past to the present and the future in response to climate change? Where were the glacial refugia for *S. cuneata* during the ice age? To answer these questions, we apply the SDMs to simulate the suitable habitat distribution of *S. cuneata* under past, present, and future climatic scenarios. We analyze changes in suitable habitat and the species responses to climate change. We also integrate fossil evidence to untangle its biogeographical history. We hope this study will provide valuable insights into the biogeographical history of *S. cuneata* and shed light on maintaining the stability of East Asian ecosystems and promoting biodiversity conservation under the scenario of global climate change.

## 2 Materials and Methods

### 2.1 Data Collection

**FIGURE 2:**
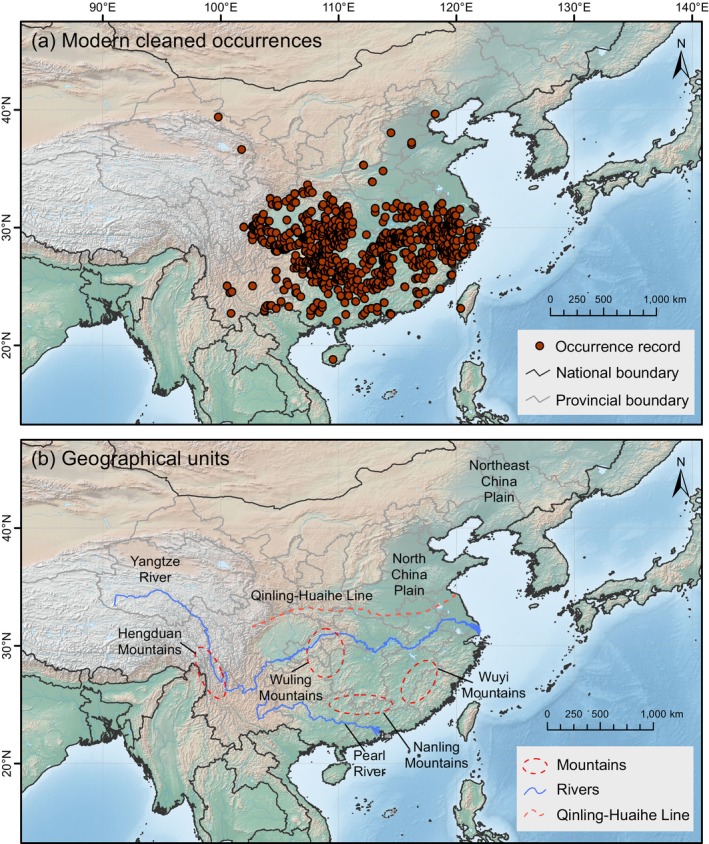
(a) Modern cleaned occurrences of *Sargentodoxa cuneata* obtained from GBIF (http://www.gbif.org) and NPSRC (http://www.cvh.ac.cn). (b) Important geographical units in China mentioned in this study include mountain ranges (indicated by red dashed circles), the Yangtze and Pearl rivers (blue lines), and the Qinling‐Huaihe Line (orange dashed line).

The modern distribution data of *S. cuneata* were obtained from the Global Biodiversity Information Facility (http://www.gbif.org, GBIF.org 2024) and the National Plant Specimen Resource Center (NPSRC, http://www.cvh.ac.cn). To ensure data quality, outliers and duplicate records were removed using CoordinateCleaner (Zizka et al. 2019) and ENMTools packages (Warren et al. 2021) in R. This ensures that a maximum of one occurrence point was retained per 5‐min grid cell. Additionally, two anomalous occurrence points from Sri Lanka and northeastern China were identified. After verification with Flora Reipublicae Popularis Sinicae (FRPS, http://www.iplant.cn) and Plants of the World Online (POWO, http://powo.science.kew.org), these two records were deemed erroneous and subsequently removed. This resulted in a total of 794 geographically valid occurrence records for the SDMs (Figure 2).

To facilitate robust species distribution modeling, 2400 pseudo‐absence points were generated. Using the disk method in the biomod2 package (Thuiller et al. 2016), pseudo‐absence points were selected within a circular buffer around presence points, with a minimum distance of 10 km.

Fossil records of the relict species *S. cuneata* play a crucial role in identifying its potential past refugia. A total of 10 fossil records were retrieved from the literature, including nine macrofossil records (e.g., Manchester 1999) primarily consisting of seeds and one pollen record in China (Song 1988). These fossil data were used to validate and disentangle the historical distribution patterns of *S. cuneata* (Table 1).

**TABLE 1:** Fossil records of *Sargentodoxa cuneata*.

Record ID	Organ type	Morphotype	Latitude	Longitude	Country	Age	References
1	Seed	*S. globosa*	44.66	−120.30	USA	Middle Eocene	Manchester (1999)
2	Seed	*S. lusatica*	51.30	12.40	Germany	Late Eocene–Late Oligocene	Mai (2001)
3	Fruit/seed	*S. cuneata*	43.83	−73.05	USA	Early Miocene (mid‐Tertiary)	Tiffney (1993)
4	Seed	*S*. sp.	31.35	−89.30	USA	Middle Miocene	McNair et al. (2019)
5	Seed	*S. lusatica*	51.50	14.20	Germany	Middle Miocene	Mai (2001)
6	Seed	*S. cuneata*	35.30	137.10	Japan	10 Ma	Momohara (2001)
7	Macrofossil	*Sargentodoxa*	36.21	−82.38	USA	Late Miocene–Early Pliocene	Mead et al. (2012)
8	Seed	*Sargentodoxa*	48.58	7.75	French	Late Miocene–Early Pliocene	Geissert et al. (1990)
9	Seed	*Sargentodoxa*	42.00	13.00	Italy	Pliocene	Martinetto (2001)
10	Pollen	*S. cuneata*	27.34	103.73	China	Plio–Pleistocene	Song (1988)

### 2.2 Environmental Variables

**FIGURE 3:**
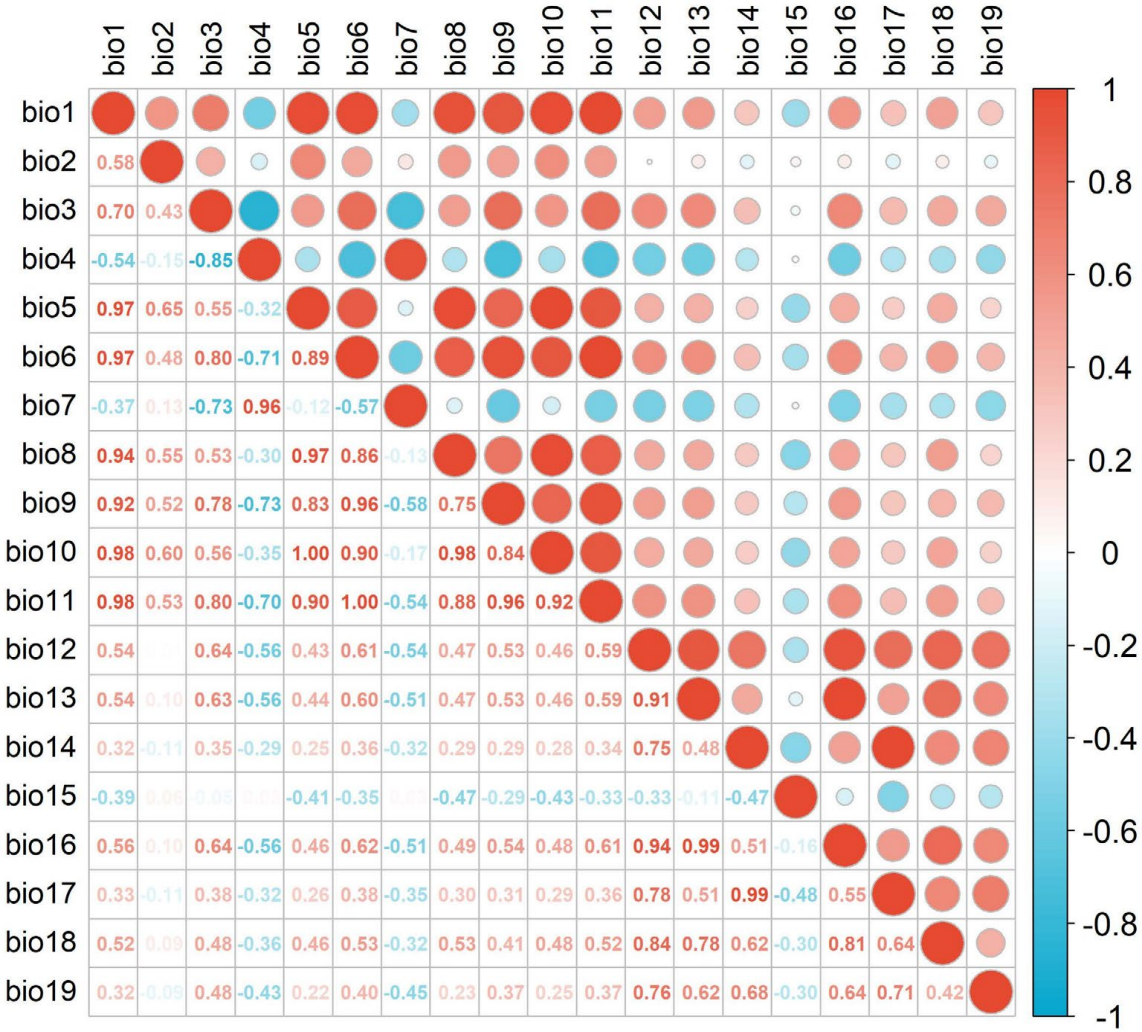
Correlation of 19 bioclimatic variables. Red and blue colors indicate positive and negative correlations, respectively. The intensity of the color and the size of the corresponding circle increase with the correlation coefficient. For example, a correlation coefficient of 0.99 between bio13 and bio16 suggests that only one of them should be retained in subsequent analyses. Abbreviations: bio1 = annual mean temperature, bio2 = mean diurnal range, bio3 = isothermality, bio4 = temperature seasonality, bio5 = max temperature of warmest month, bio6 = min temperature of coldest month, bio7 = temperature annual range, bio8 = mean temperature of wettest quarter, bio9 = mean temperature of driest quarter, bio10 = mean temperature of warmest quarter, bio11 = mean temperature of coldest quarter, bio12 = annual precipitation, bio13 = precipitation of wettest month, bio14 = precipitation of driest month, bio15 = precipitation seasonality, bio16 = precipitation of wettest quarter, bio17 = precipitation of driest quarter, bio18 = precipitation of warmest quarter, bio19 = precipitation of coldest quarter.

We selected 19 bioclimatic variables that could potentially influence the distribution of *S. cuneata* from the WorldClim Database version 2.1 (http://www.worldclim.org), with a spatial resolution of 5 min. To prevent model overfitting due to multicollinearity among environmental variables, we calculated the Pearson's correlation coefficients between variables (Figure 3). Variables showing a correlation coefficient with an absolute value less than 0.8 (|*r*| < 0.8) were retained, and this resulted in the selection of nine bioclimatic variables. Additionally, elevation data were sourced from WorldClim, and topographic factors, including slope and aspect, were calculated using ArcGIS. This led to a final set of 12 environmental variables for modeling (Table 2). These variables are ecologically meaningful for *S. cuneata* , which thrives in well‐lit, moderately humid environments and is primarily distributed in subtropical regions at mid elevations (100–1000 m) (Chen and Tatemi 2001). Consequently, climatic factors such as annual mean temperature (bio1) and precipitation seasonality (bio15), along with topographic elements such as elevation and slope, are likely to strongly influence its distribution patterns.

**TABLE 2:** Environmental factors for modeling SDMs.

Variables	Description	Units
Bio1	Annual mean temperature	°C
Bio2	Mean diurnal range (mean of monthly (max temp−min temp))	°C
Bio3	Isothermality (bio2/bio7) (×100)	/
Bio7	Temperature annual range (bio5−bio6)	°C
Bio13	Precipitation of wettest month	mm
Bio14	Precipitation of driest month	mm
Bio15	Precipitation seasonality (coefficient of variation)	/
Bio18	Precipitation of warmest quarter	mm
Bio19	Precipitation of coldest quarter	mm
Aspect	The compass direction or azimuth that a terrain surface faces	/
Elev	Elevation, height relative to datum	m
Slope	Angle of inclination of the slope	°

Future climate data were also retrieved from WorldClim v2.1, specifically from five CMIP6 climate models (ACCESS‐CM2, EC‐Earth3‐Veg, FIO‐ESM‐2‐0, MPI‐ESM1‐2‐HR, and MRI‐ESM2‐0) that have demonstrated strong performance in East Asia (Zhang et al. 2024). The ensemble mean of these models was used to project the future distribution of *S. cuneata* . Future climate projections included two time periods (2041–2060, 2081–2100) under two shared socioeconomic pathway (SSP) scenarios (SSP245, SSP585). The SSP245 scenario represents a moderate emission pathway, reflecting global efforts to mitigate climate change, while the SSP585 scenario illustrates a high‐emission pathway with limited climate mitigation measures. Additionally, paleoclimatic datasets from WorldClim v1.4 were used to reconstruct the potential historical distribution of *S. cuneata* . These datasets include climate reconstructions for the LGM and the MH (about 6000 years ago), based on the ensemble mean of three climate models (CCSM4, MIROC‐ESM, and MPI‐ESM‐P).

### 2.3 Species Distribution Models

We used the biomod2 R package (http://biomodhub.github.io/biomod2/) to develop the SDMs. A total of 10 different modeling techniques were used: artificial neural network (ANN), classification tree analysis (CTA), flexible discriminant analysis (FDA), GAM, generalized boosting model (GBM), GLM, multiple adaptive regression splines (MARS), MaxEnt, RF, and extreme gradient boosting (XGBoost). Among these, the random forest and MaxEnt models were the most frequently used. The RF algorithm aggregates the “decisions” of multiple individual trees to assign a final classification to each instance, and thereby it overcomes the limitations of a single decision tree and achieves a global optimum (Zhao et al. 2022). The MaxEnt model is a probabilistic framework based on the principle of maximum entropy, which predicts the probability distribution of species presence under given environmental conditions (Phillips et al. 2006). To optimize model performance and mitigate its complexity, we tuned two key parameters: the regularization multiplier (RM) and feature combination (FC). We tested eight RM values (0.5–4, in 0.5 increments) and five feature combinations (L, LQ, LQH, LQHP, and LQHPT) with the “ENMeval” R package (Kass et al. 2021). Here, L, Q, H, P, and T represent linear, quadratic, hinge, product, and threshold, respectively. The optimal parameter combination was determined based on the corrected Akaike Information Criterion (AICc), and the combination with the lowest delta AICc (delta.AICc = 0) was selected to achieve the best balance between model complexity and goodness of fit. Furthermore, the model's predictive performance was evaluated using the mean area under the curve (AUC) (auc.diff.avg). The final optimized settings for MaxEnt were RM = 0.5 and FC = LQHPT.

For the remaining nine models, we used the “Bigboss” parameter settings integrated in the biomod2 package, which represent empirically optimized configurations for each algorithm. This strategy has yielded equal or superior performance compared to manual tuning (Ding et al. 2025; Kim et al. 2025; Ruzzier et al. 2025). During model training, 75% of the occurrence data were randomly assigned for model training, while the remaining 25% were used for testing, with 10 iterations of random validation. Model accuracy was evaluated using two key metrics: the true skill statistic (TSS) and the area under the receiver operating characteristic curve (ROC). TSS, also known as the Hanssen‐Kuiper skill score, is a metric that integrates sensitivity and specificity (Allouche et al. 2006). The ROC curve plots the true positive rate against the false positive rate across different classification thresholds and reflects the classification ability of the model at various threshold levels. The AUC value close to 1 indicates better model performance (Shabani et al. 2018). We compared the AUC and TSS values of 10 models based on the simulation results of the modern distribution. Finally, the RF model was selected for subsequent simulations of the species suitable habitat distribution.

The final SDMs predicted the probability (*p*) of species occurrence within the study area, and this allows for the classification of suitable and unsuitable habitats. Suitable habitats were further categorized into three suitability levels—low suitability (0.2 ≤ *p* < 0.4), moderate suitability (0.4 ≤ *p* < 0.6), and extreme suitability (*p* ≥ 0.6) (Wang, Luo, et al. 2024).

## 3 Results

### 3.1 Current Distribution

**FIGURE 4:**
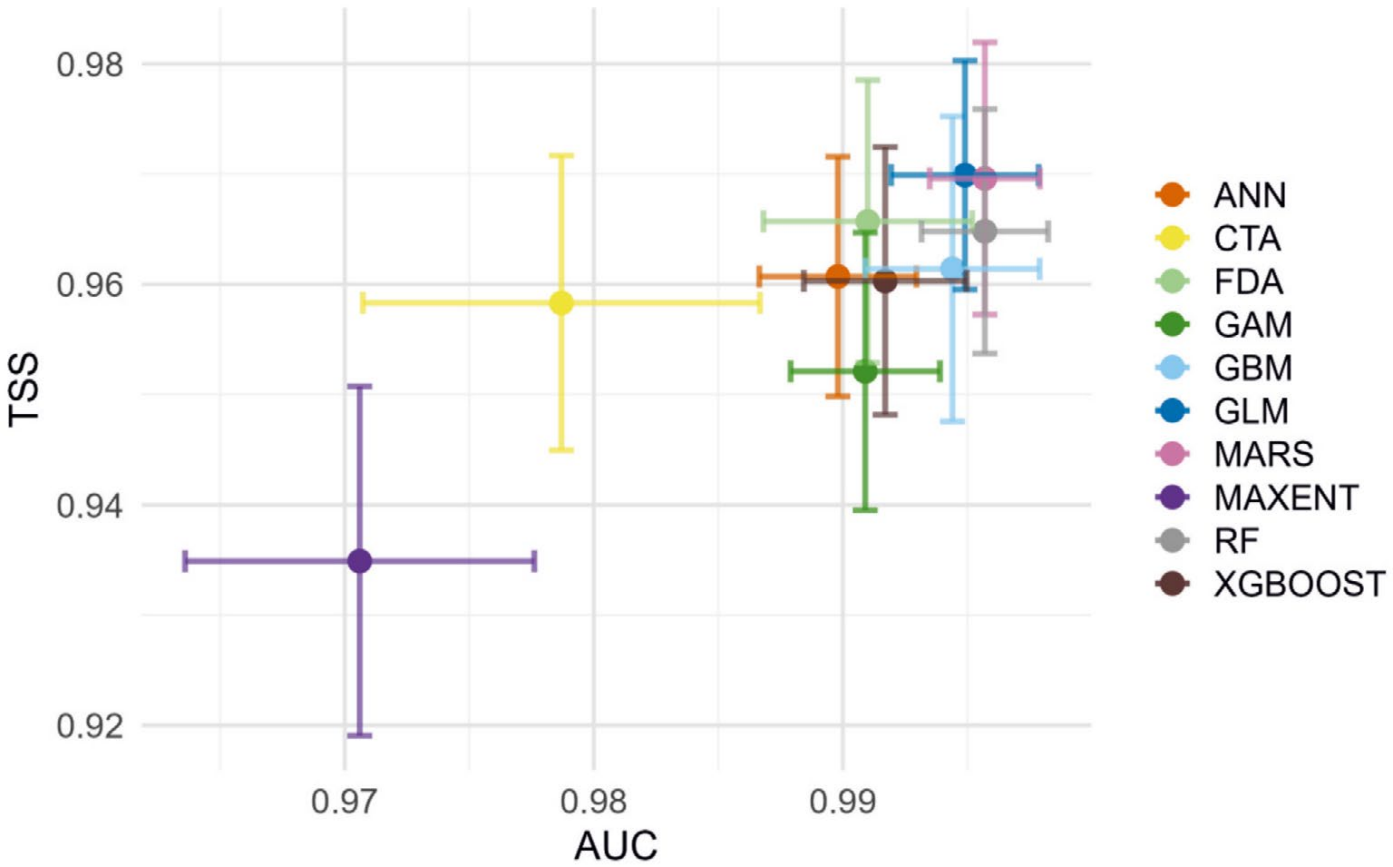
Accuracy of the ten SDM models, and their TSS and AUC values (numerical results are provided in Table S1). The error bars extending from each point represent the range of variability.

**FIGURE 5:**
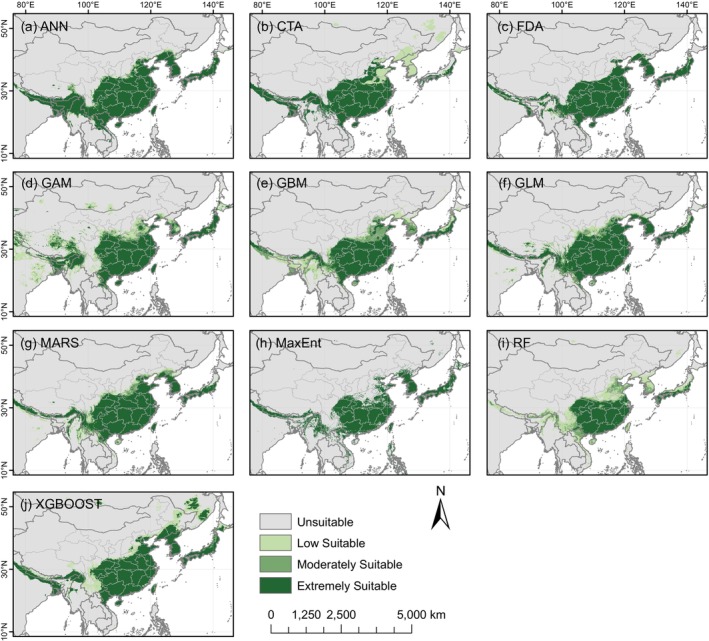
Predicted modern distribution of *Sargentodoxa cuneata* using the ten SDMs.

We used the biomod2 package to compare the accuracy of the ten SDMs (Figure 4) and their predicted species distribution results (Figure 5). Except for the MaxEnt model, the average TSS values of the remaining nine models exceeded 0.95. Similarly, with the exception of the ANN, CTA, and MaxEnt models, the average AUC values of the other seven models were above 0.99. Although most models demonstrated high predictive accuracy, there were notable differences in the distribution of suitable habitats. We found that the RF model exhibited both high accuracy and a better alignment with the actual species distribution. Moreover, it provided a more precise identification of low‐suitable and moderately suitable regions, in contrast to other models that tended to produce overly binary probability classifications (Figure 5).

**FIGURE 6:**
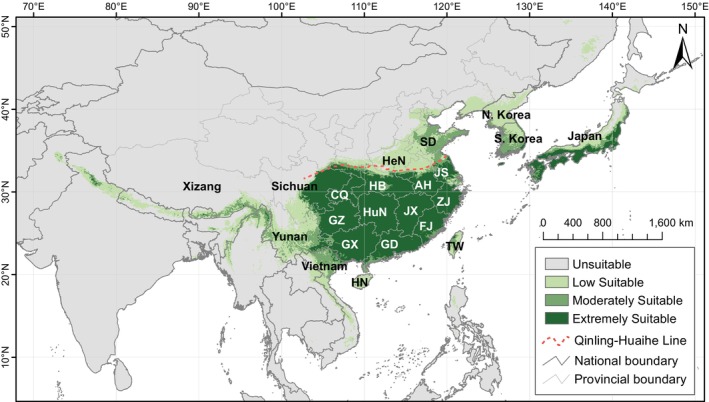
Predicted modern distribution of *Sargentodoxa cuneata* using a random forest model. Province or Autonomous Region in China: AH = Anhui, CQ = Chongqing, FJ = Fujian, GD = Guangdong, GX = Guangxi, GZ = Guizhou, HB = Hubei, HeN = Henan, HN = Hainan, HuN = Hunan, JS = Jiangsu, JX = Jiangxi, SD = Shandong, TW = Taiwan, ZJ = Zhejiang. Country: N. Korea = North Korea, i.e., the Democratic People's Republic of Korea; S. Korea = South Korea, namely the Republic of Korea.

We simulated the global distribution probability of *S. cuneata* , and found that the probability of suitable habitat outside East Asia is extremely low. Therefore, only the results for the East Asian region are presented. Under current climate conditions, the total suitable habitat area is estimated at 413.76 × 10^4^ km^2^, comprising an extremely suitable area of 177.35 × 10^4^ km^2^, a moderately suitable area of 63.06 × 10^4^ km^2^, and a low suitable area of 173.35 × 10^4^ km^2^. The species suitable habitats are primarily distributed across China and Japan. The extremely suitable areas are concentrated south of the Qinling‐Huaihe Line in China and in the southern and eastern regions of Japan. The moderately suitable and low‐suitable areas are also widely distributed in regions north of the Qinling‐Huaihe Line, including Henan and Shandong provinces, as well as Yunnan Province and the Himalayan region, Hainan and Taiwan provinces, Vietnam, the Korean Peninsula, and various parts of Japan (Figure 6).

### 3.2 Variable Importance Assessment and Response Curves

**FIGURE 7:**
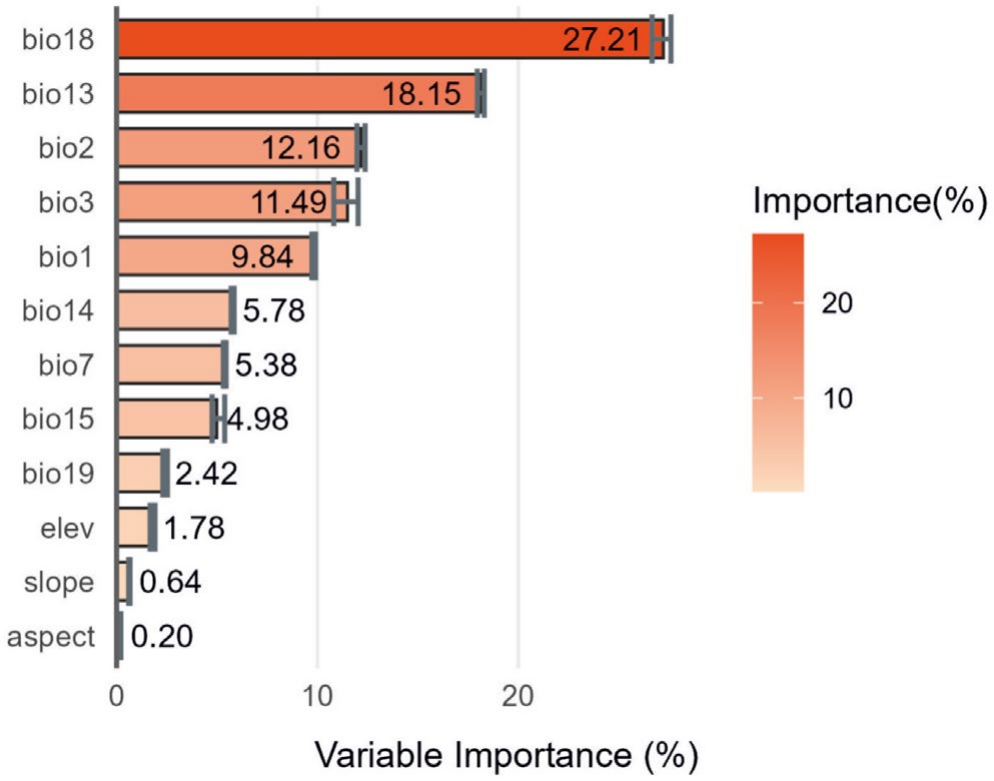
The importance of different environmental variables. The full name and unit of each variable are shown in Table 2. The gray short lines represent the fluctuation range from multiple random simulations.

**FIGURE 8:**
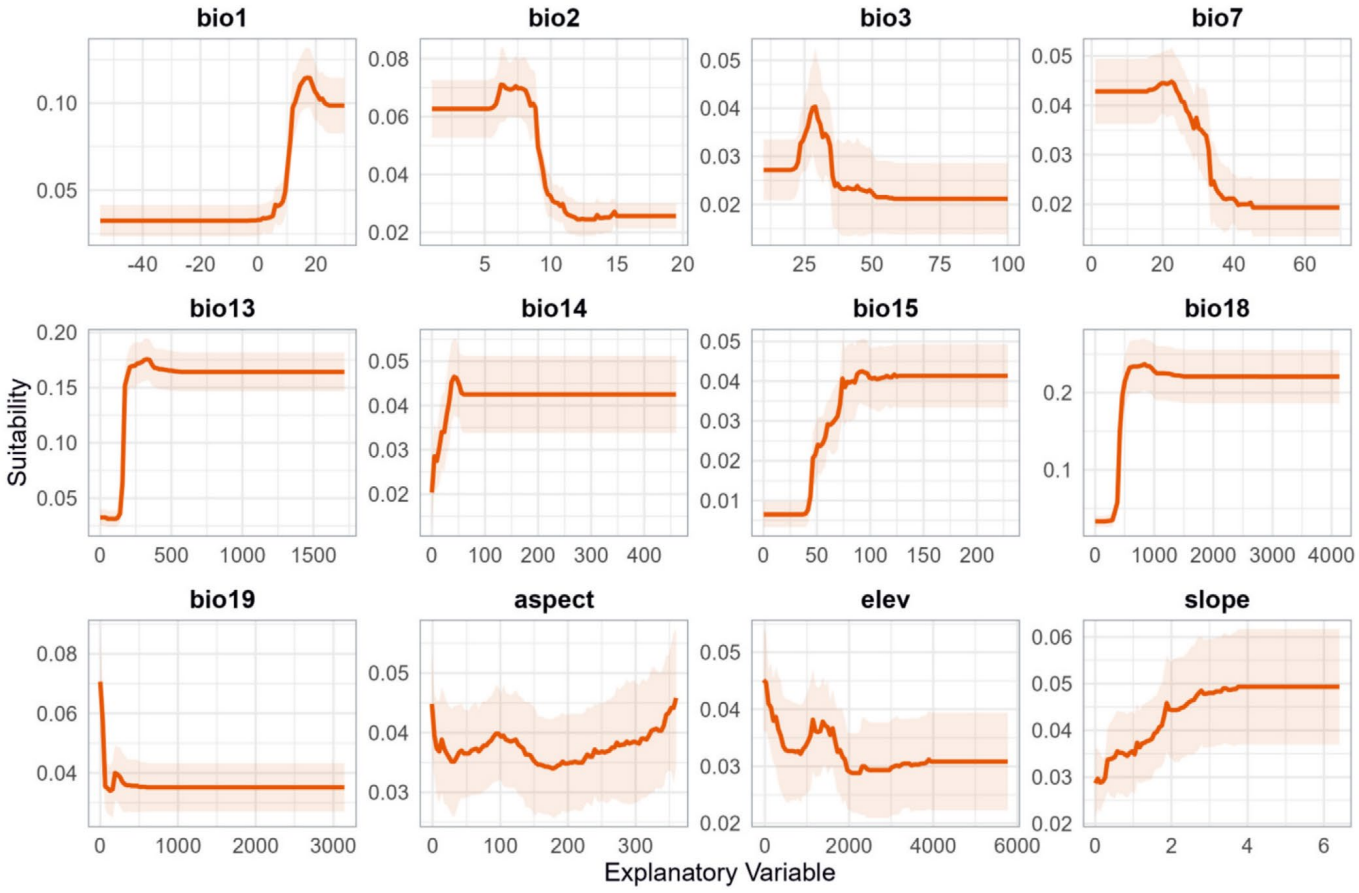
Environmental variable response curve. The full name and unit of each variable are shown in Table 2. The light red background indicates the fluctuation range of multiple random simulations.

The importance of environmental variables (Figure 7) and the response curves of SDMs (Figure 8) were analyzed to identify key factors that influence the variation in suitable habitats. Among the 12 environmental variables used in the models, the most significant factors affecting the suitable habitat distribution of *S. cuneata* were precipitation of warmest quarter (bio18), precipitation of wettest month (bio13), mean diurnal range (bio2), isothermality (bio3), and annual mean temperature (bio1). Notably, precipitation‐related factors (bio18, bio13) had a greater influence than temperature‐related factors (bio2, bio3, bio1).

The response curves of different environmental variables with respect to habitat suitability for *S. cuneata* revealed that eight variables showed clear optimal values for its growth: annual mean temperature (bio1), mean diurnal range (bio2), isothermality (bio3), temperature annual range (bio7), precipitation of wettest month (bio13), precipitation of driest month (bio14), precipitation of coldest quarter (bio18), and elevation. The most favorable conditions for *S. cuneata* growth were annual mean temperature ≈15°C, mean diurnal range ≈7°C, isothermality ≈30, temperature annual range ≈23°C, precipitation of wettest month ≈400 mm, precipitation of driest month ≈50 mm, precipitation of coldest quarter ≈750 mm, and elevation ≈0–300 m and 1500 m. Additionally, precipitation seasonality (bio15) and slope exhibited similar trends, where higher values favored *S. cuneata* growth. In contrast, the suitability of *S. cuneata* decreased with increasing precipitation of coldest quarter (bio19). Aspect showed the least consistent effect on *S. cuneata* growth and had the lowest importance among the variables. Overall, *S. cuneata* thrives in regions with moderate temperatures, high precipitation, and pronounced precipitation seasonality, particularly at mid‐ and low‐altitude.

### 3.3 Species Distribution Under Past Climatic Conditions

**FIGURE 9:**
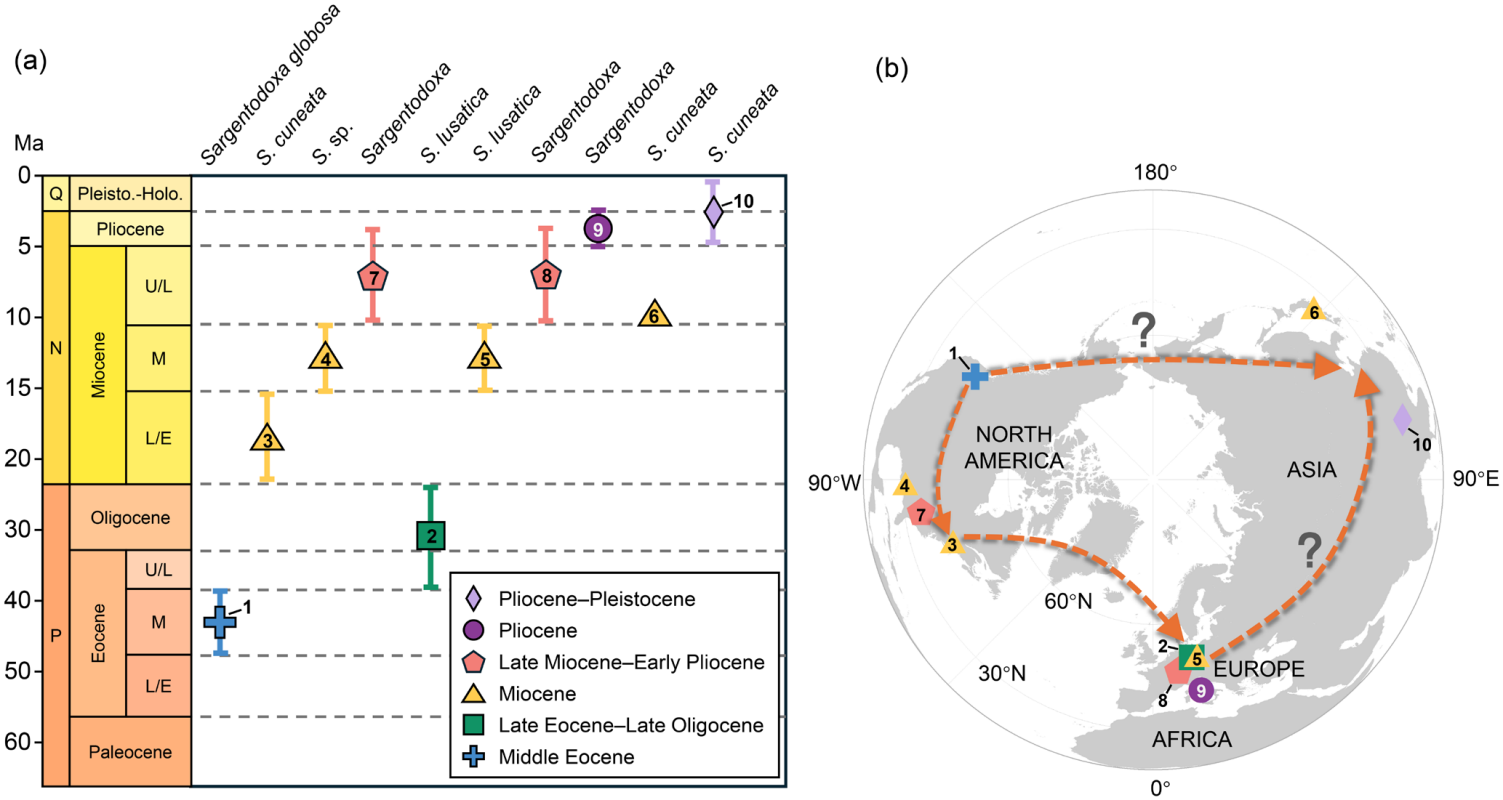
Stratigraphical ranges and global distribution, and proposed dispersal routes of *Sargentodoxa* fossils. (a) Stratigraphical ranges of *Sargentodoxa* fossil records. All fossil records are within the Cenozoic. Abbreviations: N = Neogene, P = Paleogene, Pleisto–Holo = Pleistocene–Holocene, Q = Quaternary. The short vertical lines following the symbols indicate the age uncertainty. The Miocene fossil record (no. 6) in Japan has an age of 10 Ma. Only the latest (Pliocene–Pleistocene) record (no. 10) in China is a microfossil (pollen) record, which also has an uncertain age (b) Global distribution of *Sargentodoxa* fossils across the Northern Hemisphere and possible dispersal routes. The routes with question marks indicate considerable uncertainty. The information of all fossil records is provided in Table 1. The numerical labels in the figure correspond to the Record ID in Table 1.

We mapped the fossil sites and found that the historical distribution range of *Sargentodoxa* was significantly broader than its present range. The fossil records have ages from the Middle Eocene to the Pleistocene, with occurrences in (1) the Eocene and Mio–Pliocene of North America, (2) the Eocene, Oligocene, Miocene, and Pliocene of Europe, and (3) the Miocene and Pleistocene of Asia (Geissert et al. 1990; Mai 2001; Manchester 1999; Martinetto 2001; McNair et al. 2019; Mead et al. 2012; Momohara 2001; Song 1988; Tiffney 1993) (Figure 9).

**FIGURE 10:**
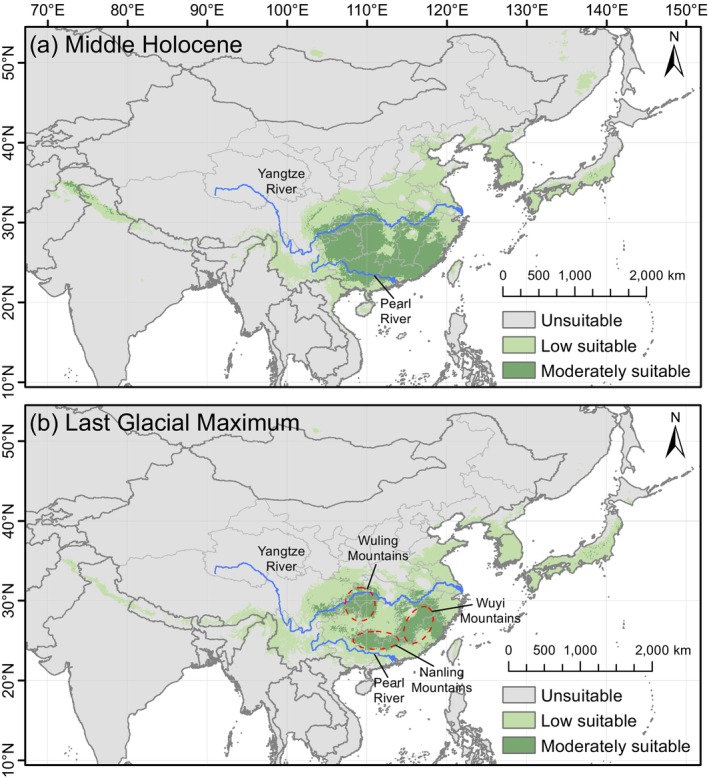
Projected suitable areas of *Sargentodoxa cuneata* in the past using a random forest model. (a) Middle Holocene; (b) Last Glacial Maximum. The red circles show the localities of Wuling, Wuyi, and Nanling mountain ranges, which played as important refugia for *S. cuneata* during the Last Glacial Maximum. The blue lines are Yangtze and Pearl rivers.

The simulations of species distribution during the LGM and MH suggest significant contractions in suitable habitat compared to the present (Figure 10). The extremely suitable area completely disappeared, while the moderately suitable area was almost entirely restricted to China, particularly to regions south of the Qinling‐Huaihe Line. The suitable habitat was more contracted during the LGM than in the MH. During the MH, the total suitable habitat area was 326.70 × 10^4^ km^2^, representing a 21.04% reduction compared to the present. The moderately suitable area expanded to 104.40 × 10^4^ km^2^, with an increase of approximately 65.55%, shifting to regions between the Yangtze–Pearl rivers, which are currently classified as extremely suitable for *S. cuneata* . In the LGM, the total suitable habitat area further decreased to 283.22 × 10^4^ km^2^, approximately 31.55% less than in the present. The moderately suitable area shrank to 56.03 × 10^4^ km^2^, with an 11.15% decline, and was primarily confined to the border of Chongqing, Hunan, and Hubei provinces (i.e., the Wuling Mountain Range), as well as the Nanling and Wuyi mountain ranges in China.

### 3.4 Future Suitable Distribution Under Climate Change

**FIGURE 11:**
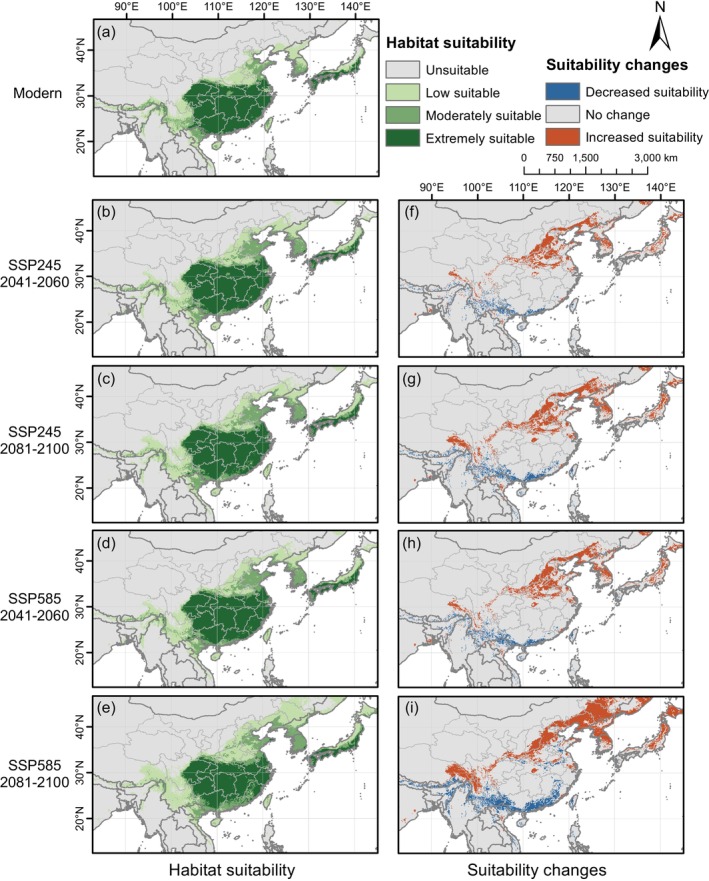
Distribution and changes of future suitable areas compared to its current distribution for *Sargentodoxa cuneata* under different scenarios. (a) Modern suitable habitat areas; (b–e) future suitable areas; (f–i) future habitat suitability changes relative to its current distribution.

The RF model was used to predict the potential suitable habitat of *S. cuneata* under the SSP245 and SSP585 scenarios for the periods 2041–2060 and 2081–2100 (Figure 11b–e). Overall, under different climate scenarios, the total suitable area is expected to increase, while the area of extremely suitable habitat is projected to decrease. Under the SSP245 scenario, the extent of change remains relatively moderate for both 2041–2060 and 2081–2100. The total suitable area is predicted to expand to 441.94 × 10^4^ km^2^ by 2041–2060 and 451.80 × 10^4^ km^2^ by 2081–2100 (Table 3). However, the extremely suitable area is projected to shrink to 174.90 × 10^4^ km^2^ and 172.10 × 10^4^ km^2^, respectively. Under the SSP585 scenario, changes are expected to be minor by 2041–2060 but become more pronounced by 2081–2100. The total suitable area will increase to 447.22 × 10^4^ km^2^ by 2041–2060, while the extremely suitable area will decline to 173.69 × 10^4^ km^2^. By 2081–2100, the total suitable area is predicted to expand to 525.11 × 10^4^ km^2^, marking a 26.91% increase compared to the present. However, the extremely suitable area is expected to decline by 11.44%, shrinking to 157.06 × 10^4^ km^2^.

**TABLE 3:** Different‐level suitable area for the present, past, and future (10^4^ km^2^), and the areal differences compared with the present suitable area (%).

Period	Extremely suitable		Moderate suitable		Low suitable		Total	
	Area	Change	Area	Change	Area	Change	Area	Change
Modern	177.35	/	63.06	/	173.35	/	413.76	/
LGM	0	−100	56.03	−11.15	227.19	31.06	283.22	−31.55
MH	0	−100	104.40	65.55	222.31	28.24	326.70	−21.04
SSP245 2041–2060	174.90	−1.38	81.69	29.55	185.35	6.92	441.94	6.81
SSP245 2081–2100	172.10	−2.96	87.03	38.02	192.66	11.14	451.80	9.19
SSP585 2041–2060	173.69	−2.07	85.09	34.94	188.44	8.70	447.22	8.09
SSP585 2081–2100	157.06	−11.44	96.67	53.29	271.38	56.55	525.11	26.91

By calculating the difference between the future and current distributions of suitable habitat, we identified regions where habitat suitability is expected to increase or decrease under different climate scenarios (Figure 11f–i). From the suitable distribution and changes in suitability, all scenarios suggest that suitability in the southern regions will decline, while northern areas will generally experience an increase. The suitability of coastal regions in southern China, most parts of Yunnan, and portions of the Himalayan region is expected to decrease, particularly under the SSP585 scenario by 2081–2100, where the decline is more pronounced. Conversely, regions north of the Qinling‐Huaihe Line, including the North China Plain, Northeast China Plain, Hengduan Mountains, eastern Xizang Province (Figure 2b), as well as central and northern Japan, are expected to experience an increase in habitat suitability. Notably, under the SSP585 scenario, by 2081–2100, the suitability of the Northeast China Plain will increase significantly.

**FIGURE 12:**
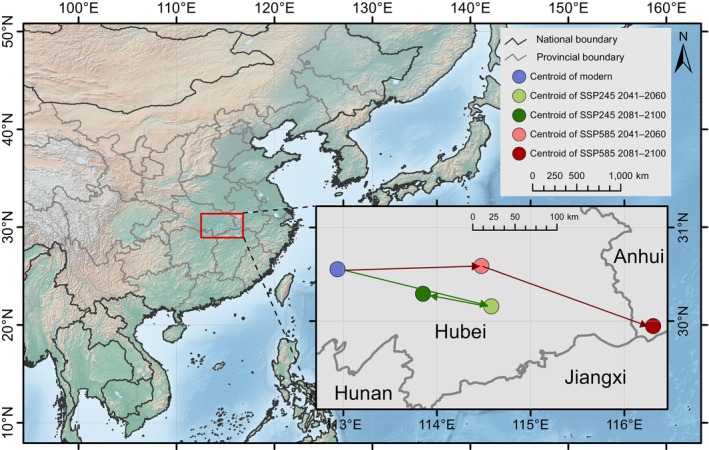
Distribution and shifts in the centroid of the extremely suitable area for *Sargentodoxa cuneata* under future climate scenarios. The green line represents movement in the path of SSP245, the red line is SSP585, and the arrow suggests the direction of movement.

Using the centroid calculation function in ArcGIS, we analyzed the shifts in the centroid of the extremely suitable area under different SSP scenarios (Figure 12). Currently, the centroid of the extremely suitable area is located in central‐southern Hubei Province (112.94° E, 30.55° N). Under the SSP245 scenario, the centroid is projected to first shift southeastward to southeastern Hubei Province (114.58° E, 30.16° N), with a displacement of 163.22 km, and then move northwestward to 113.85° E, 30.29° N, covering a distance of 71.61 km. These shifts remain within Hubei Province. Under the SSP585 scenario, the centroid is expected to first move eastward to eastern Hubei Province (114.47° E, 30.59° N), with a shift of 146.55 km, and then shift further southeast beyond Hubei Province into northwestern Anhui Province, reaching coordinates of 116.31° E, 29.94° N, after an additional displacement of 190.92 km.

## 4 Discussion

### 4.1 Model Performance Evaluation

This study compared the predictive performance of 10 SDMs implemented in the biomod2 platform to simulate the current distribution of *S. cuneata* . While all models exhibited high accuracy, their predicted geographical distributions showed noticeable differences. The observed differences in predicted species distribution probabilities may be due to the complex relationships between variables. Furthermore, each model uses different methods to define the probability of species occurrence in relation to environmental variables. Based solely on AUC and TSS values, the MARS and GLM models slightly outperformed the RF model. However, in terms of geographical distribution, RF provided results that more closely matched the actual distribution pattern. The superiority of the RF model may stem from its ability to handle complex, high‐dimensional data, particularly its capacity to effectively model nonlinear responses and mitigate the risk of overfitting (Valavi et al. 2021).

The performance of different models varies across different species (Wang, Luo, et al. 2024). Some studies have found that RF achieves the highest accuracy (Li et al. 2024; Zhao et al. 2022), whereas others report that MaxEnt outperforms other models (Cao et al. 2024; Kang et al. 2025). This highlights the inherent uncertainty across different modeling approaches and underscores the importance of selecting the most appropriate model based on the specific research objectives. Despite the strong performance of RF herein, its potential limitations should be acknowledged. Substantial biases may often arise in predictions generated by a single model under future climate scenarios. Ensemble modeling, which integrates predictions from multiple models, has been proposed as a strategy to enhance overall prediction robustness (Wang, Shi, et al. 2022; Wu et al. 2025). However, ensemble models do not always yield the best results, as an optimized single model can sometimes outperform an ensemble approach (Hao et al. 2020). Although RF performed well here, uncertainties in future predictions remain a concern, particularly under the complex dynamics of climate change. Therefore, future research could explore the use of ensemble modeling approaches while simultaneously optimizing the parameters of both single and ensemble models based on specific research objectives and data characteristics to achieve more robust and reliable predictions.

Moreover, due to the lack of available soil data for historical periods, we did not incorporate soil properties in the modeling, despite their potential to significantly influence the ecological niche of *S. cuneata* . Additionally, incomplete or uneven occurrences may reduce model accuracy by introducing spatial bias. The projections for other time periods also rely on the assumption of ecological niche conservatism (Wiens and Graham 2005). However, since ecological niches can shift over time, this introduces additional uncertainty—particularly in long‐term reconstructions such as those for the LGM and MH.

### 4.2 Potential Dispersal Routes of *Sargentodoxa*

Based on available fossil records, *Sargentodoxa* has been distributed exclusively in the Northern Hemisphere, encompassing three major regions (Europe, North America, and Asia). The earliest fossils of *Sargentodoxa* were discovered along the western coast of North America from the middle Eocene, suggesting that the genus may have originated in North America (Figure 9, Table 1) (Manchester 1999). North America is also likely the diversification center for *Sargentodoxa*. Subsequently, *Sargentodoxa* spread to Europe and Asia while continuing to disperse across North America.

It is reasonable to hypothesize that *Sargentodoxa* migrated from North America to Europe via the North Atlantic Land Bridge (NALB). There are two possible routes by which *Sargentodoxa* may have dispersed to Asia. The first route is a direct expansion from North America to Asia via the Bering Land Bridge (BLB). The second involves an initial spread to Europe, followed by an eastward migration across the Eurasian continent, eventually reaching Japan and southwestern China. However, the route across Eurasia remains highly uncertain due to the lack of fossil evidence. This migration likely occurred via the northern boreotropical route through Siberia, which provided warm‐temperate conditions during the early Tertiary and facilitated the dispersal of many thermophilic taxa. Due to the scarce fossil evidence across Eurasia, long‐distance dispersal (LDD) remains a plausible explanation. Additionally, the fleshy black berries of *Sargentodoxa*, borne on elongate pedicels, suggest bird‐mediated dispersal (Tiffney 1993), which may have enabled occasional long‐distance seed transport. Both the NALB and BLB were critical migration routes for numerous tropical, subtropical, and temperate species across the Northern Hemisphere during the Cenozoic (Hopkins 1959; Tiffney 1985). Extensive studies based on fossil records have suggested that species dispersed from North America to Europe via the NALB through Iceland (Denk et al. 2011; Jia et al. 2015; Jiang et al. 2019) and reached Asia from North America through the BLB (Wen et al. 2016). In the future, the discovery of more fossils of *Sargentodoxa* is expected to clarify its dispersal pathways and provide a more comprehensive understanding of its biogeographical history.

### 4.3 Identification of Glacial Refugia

The cyclical shifts between glacial and interglacial periods since the Quaternary have profoundly influenced modern species distributions and genetic differentiation (Hewitt 2000). Here, SDMs were used to simulate the suitable habitat of *S. cuneata* during the LGM and MH. The results indicate a significant southward contraction of the species suitable range compared to the present, with suitability levels decreasing. Notably, the extremely suitable area nearly disappeared entirely. During the LGM, global temperatures were approximately 7.0°C ± 1.0°C lower than pre‐industrial levels (Osman et al. 2021). The climate in East Asia was particularly harsh, driving tropical and subtropical species southward. As *S. cuneata* is an indicator species of warm and humid environments, its growth was severely constrained by low temperatures, leading to a more restricted distribution and reduced suitability. Consequently, compared to the MH, the suitable habitat during the LGM experienced further contraction, with the moderately suitable area also shrinking.

Based on its LGM distribution, we infer that the glacial refugia of *S. cuneata* were primarily located in the Nanling, Wuyi, and Wuling mountain ranges in China. These refugia overlap with those identified for East Asian relict species (Tang et al. 2018) and coincide with known refugial areas of the “living fossil” *Ginkgo biloba* in Southwest, East, and South China (Wang et al. 2023; Zhao et al. 2019). Among these, the Nanling Mountains (23°37′–27°14′ N) are the largest mountain range in southern China, serving as a natural biogeographical boundary for the subtropical zone. This region is recognized as a biodiversity hotspot, providing refugium for numerous relict species due to its unique topography and ecosystem (Tian et al. 2018). The Wuyi Mountains, situated in southeastern China along the border of Jiangxi and Fujian provinces, are characterized by highly complex topography, diverse habitats, and favorable climatic conditions. Due to these factors, the Wuyi Mountains have been widely recognized as a natural gene bank for biological species (Chen et al. 2024). This makes their role as a refugium for *S. cuneata* unsurprising. The Wuling Mountains, a northeast‐southwest trending mountain range in central China (27.28°–30.05° N, 107.02°–111.33° E), exhibit complex and diverse vegetation and serve as a biodiversity hotspot for plant species in central China (Sun et al. 2024; Wang, Zhou, et al. 2022). This region has also been identified as a refugium for many ancient plant species in China (Qi et al. 1998). The orientation of mountain ranges is closely related to the direction of species dispersal (Xiao et al. 2023). The mountain ranges that served as glacial refugia for *S. cuneata* generally follow north‐south, east‐west, and northeast‐southwest directions. These mountains facilitated its post‐glacial dispersal across southern China and even East Asia.

Fossil evidence further reveals that *Sargentodoxa* had a much wider distribution in the Paleogene (~40 Ma) and Neogene (~15 Ma), with a global presence. However, since the Quaternary (~2.58 Ma), its distribution has become restricted to China (Figure 9, Table 1). This pattern suggests that *Sargentodoxa* experienced significant geographical contraction not later than the Quaternary, likely associated with climatic fluctuations and environmental changes. The existence of multiple, spatially dispersed glacial refugia likely enabled the species to persist in small populations during harsh glacial‐interglacial cycles. These refugia facilitated postglacial range expansions across the Chinese subtropics, gradually shaping its current restricted distribution (Tian et al. 2015). Given their critical role in sustaining biodiversity and ecosystem stability, these refugial regions warrant prioritized conservation efforts.

### 4.4 Impact of Climate Change on Its Distribution

The analysis of species response curves to environmental factors sheds light on the understanding of the appropriate distribution of species under future climate change and the intrinsic reasons for the changes. Analysis of the species response curves to environmental variables revealed that precipitation plays a slightly more critical role than temperature. As a climbing plant species, *S. cuneata* has high water requirements, and sufficient precipitation directly influences its growth and reproductive success. Numerous studies have identified water availability as a key limiting factor for liana growth (e.g., Jiang et al. 2011). Additionally, the response curve of precipitation seasonality (bio15) exhibits a strong linear relationship, indicating that *S. cuneata* thrives in environments with pronounced seasonal precipitation variation. This suggests that the species has effectively adapted to the distinct seasonal rainfall patterns associated with the East Asian monsoon climate. The ample precipitation in these regions provides the moist environment necessary for its growth (Ding and Johnny 2005). Furthermore, researchers observed a “midday depression” phenomenon in the photosynthesis of *S. cuneata* leaves during noon hours of summer, indirectly supporting our finding that the species' suitability declines when temperatures exceed a certain threshold (Jin et al. 2002). Interestingly, the suitable altitude range for *S. cuneata* may exhibit two distinct intervals: a lower altitude range (0–300 m) and a mid‐altitude range (around 1500 m). Among these, the lower altitude range appears to be more suitable than the mid‐altitude range, although this observation lacks sufficient literature support. Additionally, within the lower altitude range of approximately 0–500 m, habitat suitability decreases with increasing altitude. A possible explanation is that *S. cuneata* is more easily discovered at lower altitudes, leading to a higher number of occurrence records compared to those at mid‐altitudes, which introduces bias. Future studies should incorporate a more comprehensive set of occurrence points and consider the physiological characteristics of *S. cuneata* to further refine the analysis of its suitable altitude range. Overall, *S. cuneata* is best adapted to mid‐ and low‐altitude regions with moderate temperatures, abundant precipitation, and distinct precipitation seasonality.

The response curves of environmental factors indirectly confirm that mountainous regions have been, and will continue to be suitable habitats for *S. cuneata* . Mountains provide diverse topographies that offer a wide range of ecological niches, ensuring species survival across various environmental conditions. Moreover, mountain uplift influences atmospheric circulation and provides abundant orographic precipitation. The diversity of soil types and the abundance of water resources in mountainous regions further enhance the availability of essential survival resources for species. In China, the east‐west and northeast‐southwest oriented mountain ranges also block cold air from moving southward. For instance, the climate south of the Qinling‐Huaihe Line is warmer and more humid. These unique geomorphological and ecological characteristics make mountains important refugia for numerous species, which play a critical role in maintaining global biodiversity during glacial‐interglacial cycles (Antonelli et al. 2018; Hoorn et al. 2013). For example, the Hengduan Mountains in China have preserved a wide range of endemic and relict plant species by providing stable habitats (Sun et al. 2017). This highlights the vital role that mountainous refugia play in biodiversity conservation.

According to IPCC projections, by 2100, global surface temperatures are expected to rise by 2.7°C under the moderate‐emission SSP245 scenario and by 4.4°C under the high‐emission SSP585 scenario (Intergovernmental Panel on Climate Change [IPCC] 2021). Our predictions of future habitat suitability indicate that under the SSP245 scenario, the species suitable habitat will not differ significantly from its current distribution, with a general northward shift. However, under the SSP585 scenario, the suitable range will undergo a more pronounced northward expansion. Climate warming is identified as the primary driver of *S. cuneata* 's northward expansion and southern range contraction. These findings have important implications for the conservation of *S. cuneata* , a relict species with high medicinal value. Future conservation efforts should prioritize mountainous regions in southern and central China, which are projected to remain climatically suitable. Additionally, the modeling approach used in this study may serve as a valuable reference for assessing the impacts of climate change and developing conservation strategies for other relict or climate‐sensitive medicinal plants in East Asia.

## 5 Conclusions

In this study, we compared nine machine learning models and the MaxEnt model to assess their predictive performance, ultimately selecting the RF model for simulating the past, present, and future suitable habitats of *S. cuneata* under climate change scenarios. The results indicate that *S. cuneata* thrives in mid‐ and low‐altitude regions with moderate temperatures, abundant precipitation, and distinct precipitation seasonality, with its extremely suitable habitats primarily located south of the Qinling‐Huaihe Line in China. Precipitation‐related factors had a greater influence on distribution than temperature‐related factors. During the LGM and MH, the species suitable range contracted significantly, with reductions of 130.54 × 10× km² and 87.06 × 10× km^2^, respectively. The contraction was most pronounced during the LGM, when its suitable habitat was restricted to the Nanling, Wuyi, and Wuling mountain ranges—potential glacial refugia for the species. By 2100, model simulations indicate that under two different scenarios (SSP245 and SSP585), the total area of suitable habitat is expected to expand, with a noticeable trend of northward expansion and a corresponding contraction in the south. While the centroid of highly suitable habitats is expected to shift slightly eastward. As a representative relict plant species in China, *S. cuneata* shares similar responses to climate change and glacial refugia with other South Asian relict species. This study will place an important basis for future conservation strategies and climate adaptation measures for relict plants and thus contribute to biodiversity conservation and sustainable ecosystem management. Future research combining more comprehensive fossil data, genetic analysis, and ecological modeling can further elucidate the evolutionary history, dispersal, and geographic distribution of *S. cuneata* .

## Author Contributions

**Xuanqi Liu:** conceptualization (equal), data curation (lead), investigation (equal), methodology (lead), validation (lead), visualization (lead), writing – original draft (lead), writing – review and editing (supporting). **Huasheng Huang:** conceptualization (lead), investigation (equal), methodology (supporting), project administration (lead), supervision (lead), writing – review and editing (lead). **Xia Meng:** validation (supporting), visualization (supporting), writing – review and editing (supporting). **Minqiao Li:** validation (supporting), visualization (supporting), writing – review and editing (supporting). **Zeyu Qin:** visualization (supporting), writing – review and editing (supporting).

## Ethics Statement

The authors have nothing to report.

## Consent

The authors have nothing to report.

## Conflicts of Interest

The authors declare no conflicts of interest.

## Supporting information


**Table S1.** TSS and AUC values of the ten SDM models.

## Data Availability

All data generated or analyzed during this study are included in this published article.
