# Supplementary material for: The Past, Present, and Future Distribution of Sargentodoxa: Perspectives From Fossil Record and Species Distribution Models
Source: Ecol Evol. 2025 Jul 20;15(7):e71831. doi: 10.1002/ece3.71831 (PMC12277121; doi:10.1002/ece3.71831)
Supplement: Supplementary file 1 — Table S1. TSS and AUC values of the ten SDM models. [file ECE3-15-e71831-s001.docx]

Supporting Information for “*The past, present and future distribution of* Sargentodoxa*: Perspectives from fossil record and species distribution models*” by Liu et al.

**Table S1.** TSS and AUC values of the ten SDM models.

| SDM models | ROC | TSS |
| --- | --- | --- |
| ANN | 0.9898 | 0.9607 |
| CTA | 0.9787 | 0.9583 |
| FDA | 0.991 | 0.9657 |
| GAM | 0.9909 | 0.9521 |
| GBM | 0.9944 | 0.9614 |
| GLM | 0.9949 | 0.9699 |
| MARS | 0.9957 | 0.9696 |
| RF | 0.9957 | 0.9648 |
| XGBOOST | 0.9917 | 0.9603 |
| MAXENT | 0.9706 | 0.9349 |
